# Design of a Mobile App Interface That Engages Community Members in a Food System Pilot Study

**DOI:** 10.3390/nu16111723

**Published:** 2024-05-31

**Authors:** Emma C. Lewis, Stacey Williamson, Yutong Xie, Lisa Poirier, Ayoyemi T. Oladimeji, Takeru Igusa, Joel Gittelsohn

**Affiliations:** 1 Department of International Health, Johns Hopkins Bloomberg School of Public Health, Baltimore, MD 21205, USA; swill363@jh.edu (S.W.); lpoirie4@jh.edu (L.P.); jgittel1@jhu.edu (J.G.); 2 Department of Epidemiology, Johns Hopkins Bloomberg School of Public Health, Baltimore, MD 21205, USA; yxie62@jh.edu; 3 Department of Civil and Systems Engineering, Johns Hopkins Whiting School of Engineering, Baltimore, MD 21218, USA; toladim3@jh.edu (A.T.O.); tigusa1@jh.edu (T.I.)

**Keywords:** urban, food access, corner stores, digital health, mHealth, mobile app, community engagement

## Abstract

Supermarkets are scarce in many under-resourced urban communities, and small independently owned retail stores often carry few fresh or healthy items. The Baltimore Urban food Distribution (BUD) mobile application (app) was previously developed to address supply-side challenges in moving healthy foods from local suppliers to retailers. In-app opportunities for consumers to indicate demand for these foods are crucial, but remain absent. We sought to understand community members’ perspectives on the overall role, function and features of a proposed consumer-engagement module (BUDConnect) to expand the BUD app. A series of initial high-fidelity wireframe mockups were developed based on formative research. In-depth interviews (*n* = 20) were conducted and thematically analyzed using ATLAS.ti Web. Participants revealed a desire for real-time crowd-sourced information to navigate their food environments safely and effectively, functionality to help build community and social networks among store owners and their customers, opportunities to share positive reviews and ratings of store quality and offerings, and interoperability with existing apps. Rewards and referral systems resulting in the discounted purchasing of promoted healthy items were suggested to increase adoption and sustained app use. Wireframe mockups were further refined for future development and integration into the BUD app, the program and policy implications of which are discussed.

## 1. Introduction

Obesity and its correlates are highly prevalent across the United States, especially impacting under-resourced urban communities where residents are more likely to have lower incomes and belong to racial/ethnic minority groups [1]. It is well documented that obesity substantially increases the risk of other chronic diseases (e.g., hypertension, diabetes, and cancer), all of which have been linked to poor dietary quality [2]. The diets of under-resourced urban adults tend to include less nutritious foods (e.g., high-fat, -sodium, and -sugar prepared and packaged items) and beverages (e.g., sugar-sweetened drinks), and often fail to meet the recommended amounts of fruits, vegetables and dietary fiber [3,4,5]. Taken together, these patterns may culminate in adverse health outcomes.

Although there are numerous causes of obesity, challenges within the food system are a primary driver [6]. In communities where supermarkets and other healthy food sources are scarce, energy-dense nutrient-poor processed foods are easily acquired at small retail stores (e.g., corner and convenience stores). Previous interventions have successfully utilized existing small retailers as venues for increased access to and promotion of healthier options [7,8,9,10]. However, in Baltimore, Maryland, corner store owners face several barriers to maintaining stocks of affordable healthy foods and beverages. These include high wholesaler minimum purchase requirements and delivery costs that are unattainable relative to their store’s space and profit margin. Instead, corner store owners typically must obtain, transport, and resell items such as fresh produce, milk, and whole-wheat bread from big-box stores—a burdensome process that may not be perceived as worthwhile without apparent consumer demand.

Nevertheless, formative research in this setting has revealed impaired communication between corner store owners and their customers due to cultural and language barriers. Most corner store owners in Baltimore do not come from or live within the communities their stores serve, with the majority having distinct cultural backgrounds (an estimated 60% Korean American, 20% Chinese American, and 5% Hispanic American) from their customers (64% African American). This can hinder engagement within the store setting, and may influence retailer and consumer perceptions and behavior [11]. In fact, a prior study in Baltimore revealed that corner store owners believed their customers were uninterested in purchasing and consuming healthy foods, despite interviews with corner store shoppers demonstrating otherwise [12,13,14]. In Washington, D. C., surveyed corner store owners voiced strong risk aversion regarding stocking healthy products in their stores without a demonstrated demand for those items [15].

Therefore, a platform to facilitate engagement between corner store owners and consumers while accommodating language and cultural barriers is warranted. This sentiment aligns with the Centers for Disease Control and Prevention’s prioritization of actively involving community residents, or consumers, in research for increased empowerment and capacity building to obtain better health conditions (e.g., improved healthy food access) [16]. Digital strategies have the potential to effectively integrate community-based research into systems-based interventions given the ubiquity and power of tools such as smartphones [17]. User-centered mobile applications (apps) in particular harness the reach and connectivity necessary to allow for voices to be heard equitably, and to source big data to inform future programs and policies [17]. However, user acceptance of mobile app technology is closely linked to the success or failure of the technology, thereby making it crucial to ensure apps are designed to adequately support the needs of intended users in their real-world environments [18].

Previously, we developed the Baltimore Urban food Distribution (BUD) app to move fresh, healthy foods from local suppliers to corner store owners in Baltimore via in-app collective purchasing and shared delivery [19,20]. Although robust in its ability to mitigate supply-side challenges for retailers, the app does not currently offer consumer engagement or input opportunities to indicate demand. In order to fill this gap, we sought to develop a consumer-based module (‘BUDConnect’) to optimize the BUD app’s supply–demand feedback loop for corner store stocking and purchasing of healthy foods based on the Retail Food Environment and Customer Interaction Model [16]. The model depicts reciprocal relationships between consumers and their retail food environments, subsequently influencing the stocking and sales of items, within a multilevel context that accounts for consumers’ and retailers’ characteristics and interactions. Therefore, our guiding research questions included the following:

1. What do community members perceive to be the potential role(s) of an app for local food sourcing in their neighborhoods?
2. How do community members react to the function and features of the initial BUDConnect user interface wireframe mockups, and how can the interface be improved for future development and testing?

## 2. Materials and Methods

### 2.1. Setting

The present study took place in Baltimore, Maryland, where small independently owned corner and convenience stores amounted to over 700 locations (525 corner stores, 183 convenience stores) citywide prior to the pandemic [21]. An estimated one quarter (23.5%) of residents live in areas deemed “Healthy Food Priority Areas” (e.g., food deserts or swamps), and are disproportionately more likely to be of racial/ethnic minority backgrounds than white [21]. Despite their low median household incomes, most (81.9%) households in Baltimore have smartphone access [22].

### 2.2. Participants

Participants (*n* = 20) were adult consumers who self-identified as regular shoppers of a corner store(s) in Baltimore. Recruitment occurred via word-of-mouth (e.g., snowball sampling) and social media marketing within our existing networks. Data saturation began to be reached around the fifteenth participant, and was fully reached by the twentieth.

### 2.3. Study Design

Interested community members were asked to email a project-dedicated Google Gmail account, at which point the research team communicated using a series of scripted emails to schedule an interview via Zoom. Participants were required to have their audio and video turned on for the entirety of the Zoom call. Two trained research team members (E.C.L., S.W.) attended all Zoom calls and took turns serving as the primary interviewer, or the note-taker. Once participant eligibility was confirmed and oral consent was provided, the primary interviewer began the semi-structured in-depth interview process, which lasted approximately 30 min. Zoom cloud recording was utilized to generate initial transcriptions and Descript web-based software was used to refine transcriptions line-by-line. Recordings, transcripts and notes were de-identified and stored in a secure Johns Hopkins OneDrive folder. At the end of the interview, participants received a USD 30.00 Amazon e-gift card and were asked to share the recruitment flyer with local friends, family and colleagues. All research activities were approved by the Johns Hopkins Bloomberg School of Public Health Institutional Review Board (IRB #00017307).

### 2.4. Data Collection

Interviews began with the primary interviewer providing a brief overview of the BUD app and proposed BUDConnect module. Once the participants understood the background context, they moved onto the semi-structured in-depth interview guide. The research team developed the guide, which comprised three parts, based on a data collection instrument used in a previous study to gather formative research for the development of the BUD app [23].

**Figure 1:**
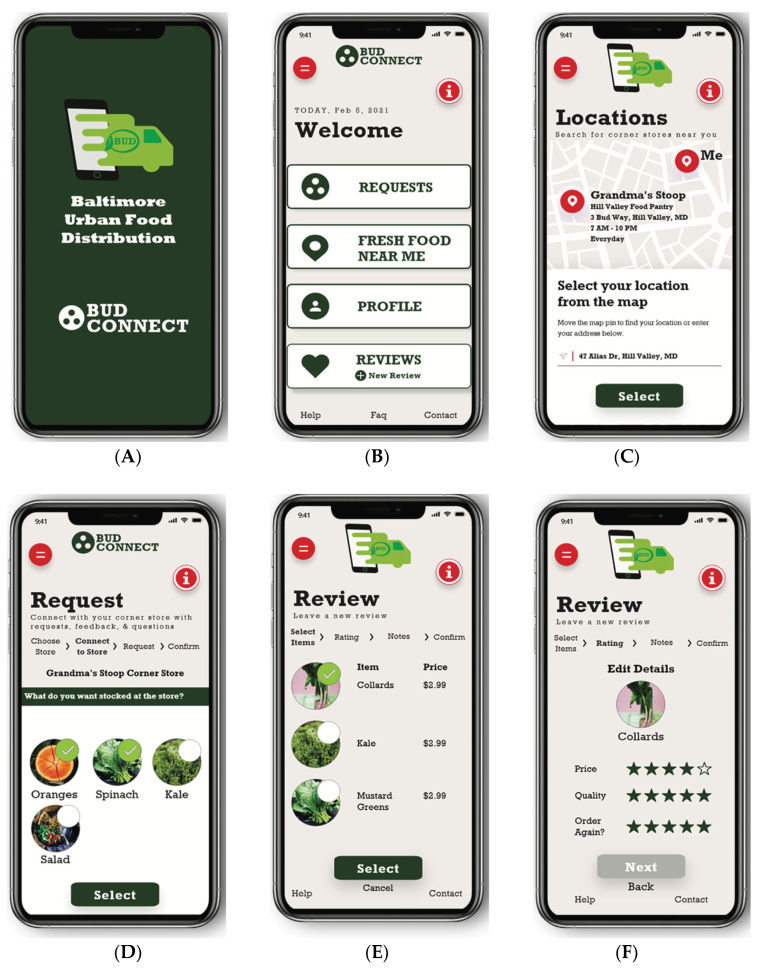
Initial BUDConnect wireframe mockups shown to interview participants. (**A**) Splash Screen, (**B**) Welcome Page, (**C**) Interactive Map, (**D**) Requests, (**E**) Reviews, (**F**) Ratings.

The first part contained 12 open-ended questions with probes pertaining to the participants’ neighborhood and community, familiarity with app use among their community, experiences shopping within their local food retail environments, perceived community needs and initial thoughts or reactions to the concept of BUDConnect. The second part contained 10 closed-ended questions aimed at gathering sociodemographic information, including the United States Department of Agriculture (USDA) 2-Item Food Security Screener [24], food assistance program enrollment and individual- and household-level characteristics (e.g., age, gender, race/ethnicity, marital status, education and employment and household annual income). Finally, the third part involved displaying six high-fidelity wireframe mockup images (Figure 1) of a BUDConnect user interface to elicit immediate reactions and specific feedback. The research team developed these initial wireframe mockups in two prior steps: (1) identification and content mapping of reasonable app features guided by formative research and a landscape analysis of existing consumer apps; and (2) depiction of an intuitive app interface based on a prototype that was previously generated using Adobe software products (e.g., Photoshop 25.0-25.8, Illustrator 28.0-28.5 and XD 2024) and usability tested in this setting [20,25]. At this stage, the primary interviewer also described the intended goals of several proposed features, including the following:

1. An interactive map (Figure 1C) to allow users to locate nearby corner store addresses, hours of operation, currently stocked healthier foods and ongoing promotions or deals in real-time.
2. Collective feedback (Figure 1D,F) to crowd-source community insights and encourage store owners to maintain stocks of affordable, fresh, healthy products via polls, chat messaging and reviews or ratings (e.g., store cleanliness and available variety, quality and price).
3. Built-in gamification to enhance the user experience and encourage sustained app use, such as a point system to reward engagement.

Participants were probed on likeability of content and display, missing features or gaps and aspects of feasibility (e.g., acceptability, operability and perceived sustainability), with themselves and other members of their communities in mind. Once the interview was completed, the primary interviewer and note-taker conducted peer debriefing and assessed the current level of data saturation.

### 2.5. Data Analyses

Interviews resulted in both quantitative and qualitative data. Quantitative data (e.g., socio-demographics) were entered and analyzed using Microsoft Excel for Mac (version 16.81) [26]. Qualitative data were derived from transcriptions and notes and coded using a hybrid deductive/inductive thematic analysis approach [27,28]. Following familiarization with the data, the coding process began with initial deductive code generation by the two primary coders (E.C.L., S.W.) and subsequent inductive code refinement. Themes were then generated, defined and named. The resulting codebook underwent analytic triangulation with a third trained qualitative researcher (Y.X.) who aided in the review of themes and sub-themes for enhanced reliability before application to the data. Finally, the identification and organization of exemplars for each theme and sub-theme occurred in ATLAS.ti Web (version 24) by all three coders (E.C.L., S.W., Y.X.) [29].

## 3. Results

### 3.1. Characteristics of the Study Sample

Participants ranged in age from 24 to 54 years old (mean = 36.85 years), and the majority were female (65%). A total of 30% identified as Black or African American, 45% reported experiencing food insecurity in the past 12 months and 40% estimated having an annual household income of less than USD 20,000. Fourteen distinct Baltimore neighborhoods were represented. Table 1 depicts participant sociodemographic characteristics.

### 3.2. Community Member Perspectives

Participants generally reported a high level of acceptability for the BUDConnect concept and initial interface. Based on our thematic analysis and guiding research questions, five key themes (Table 2) were identified: (1) the app could make food sourcing safer and more successful, (2) the app could help build community, (3) the app could support the resurgence of “corner store culture”, (4) the app could help consumers make informed food source choices and (5) app form and function suggestions.

### 3.3. Perceived Roles of an App for Neighborhood Food Sourcing

Themes 1–4 informed an understanding of the various roles that community members perceived a corner store food sourcing app could play in their neighborhoods, given their lived experiences.

Theme 1: The App Could Make Food Sourcing Safer and More Successful

When asked to describe their neighborhoods, participants commonly mentioned aspects such as urbanicity—including walkability and proximity to retailers from their residences—as well as safety and crime, and types of food sources available to them. The ability to access various food sources impacted how participants felt an app like BUDConnect may or may not serve them. For example, one participant shared,

*“It’s not like the most safe of neighborhoods. It’s hard to feel like I can venture everywhere.”*

—35 year old Middle Eastern female

This participant further noted that if an app could locate nearby corner stores and provide a suggested walking route, she may feel more confident in her ability to access those stores safely and quickly instead of walking block-to-block to search for them independently. It is important to note that, in this setting, corner stores often do not have an online presence and rarely appear on existing tools such as Google Maps or Apple Maps—if they do, the store address, hours of operation and business name tend to be outdated.

Another participant who lacked personal transportation shared that knowing the current stocking of nearby food sources would be helpful, especially when she is in search of a particular item,

*“Maybe I want carrots, and they only have, like, a bag, and they sell out [quickly], I think it would be very convenient just to know they have that.”*

—36 year old Hispanic female

Other participants, regardless of whether they could travel outside of their neighborhoods, often mentioned that a benefit to living in the city was the proximity to various retailers—and they would prefer to shop locally if they had a better idea of which stores to go to, how to get there and what is currently stocked. Without having this information readily available, several participants shared about the experience of walking around their neighborhoods to source what is needed, having to,

*“…hop from store to store to try to find the items, like, if you don’t have time to go to the market. And you’re just missing maybe two items from a meal and you go to the corner store, you’d have to go to maybe two or three corner stores and try to find those two items.”*

—43 year old American Indian female

Similarly, another participant shared,

*“A lot of times you just go there because you’re like, I want that one thing [and] I got to go real quick. And then like they don’t have it so you go to the next one a block away and they don’t have it so then you got to go four blocks to the grocery store and this whole thing was just to like, save a four block walk.”*

—33 year old White male

Although one participant described small stores in their neighborhood as offering everything a grocery store would have, most others had trouble finding fresh, healthy items besides the occasional single piece of fruit (e.g., a banana or orange). In some instances, the presence of neighborhood food pantries and food banks, including those run by local church organizations, seemed to help supplement participants’ access to resources and food.

Theme 2: The App Could Help Build Community

Participants regarded community cohesion as an indicator of neighborhood culture, and the needs of various community members were underscored as important considerations for the app design and development. One participant shared,

*“Clearly there’s a need for fresher produce and things like dairy for people in the community…[having an app] would be probably a lot better for the community, it’d save a lot of time. I wouldn’t have to hop from corner store to corner store to find two items…especially when you’ve got two little ones at home and dinner to prepare.”*

—43 year old American Indian female

Another participant noted that a tool like BUDConnect could,

*“…just help really build community, if it’s something that would be engaging to all of our neighbors.”*

—27 year old Asian non-binary person

This participant added that many of their neighbors are older and unable to walk far or for long periods of time to source food, and could benefit from being able to use an app to find a nearby store with the items they need.

Community cohesion and needs were also discussed in the context of the cultural and ethnic characteristics of specific neighborhoods in Baltimore, such as Highlandtown, Greektown and Little Italy. For example, one participant living in Little Italy noted that,

*“It’s a big Italian community, and also it’s a Jewish community as well. It’s the kind of community where you want to go and walk around because there’s a lot of history here…if you build a relationship with people in the community, you’re going to see a trend where everybody’s coming in [to local stores].”*

—52 year old White male

In addition, another participant shared,

*“Each community is different. And with so many communities in Baltimore City, it’s important to have a good representation of each to service them best…the demands are different for certain foods.”*

—43 year old American Indian female

There was interest in exploring the possibility of the app as a social platform for community members to share pictures of the meals they cook with foods purchased from corner stores. In addition, several participants suggested that users might earn points or rewards for recommending the app to others, thus creating a “win–win” situation for app users and their peers.

Theme 3: The App Could Support the Resurgence of “Corner Store Culture”

In addition to highlighting the culture within their neighborhoods, participants also emphasized the importance of amplifying the culture within store retail environments, including the dynamics between store owners and their customers. Several participants noted being familiar with the owner of their local store, and these same participants tended to feel as though store owners cared for the communities they served. For example, one participant shared about the owner of the store below her apartment building, noting that,

*“He will often ask, are you looking for something? So I think, you know, he’s a nice guy and cares about the customers. I often see him greeting everybody.”*

—36 year old Hispanic female

Others had similar experiences, yet still felt uncomfortable confronting store owners with questions or requests,

*“The guy that runs that store, you know, we get along well. But, it’s like, I don’t know what you’re doing to stock the shelves. And I’m not going to ask questions.”*

—32 year old White female

On the other hand, some participants shared experiences such as,

*“I go in [the store] for what I need and leave.”*

—30 year old Black female

*“I’m kind of just in and out because I don’t go in there to like browse so much as like, I go in there needing something and I leave relatively quickly.”*

—29 year old Asian female

Regardless, participants had certain expectations as to how willing store owners would be to make requested changes. One participant highlighted a nearby store where she felt that the owner would not be motivated to improve their store’s stock,

*“They don’t seem like they care enough if the community has healthy stuff…it’s more of a for-profit situation.”*

—38 year old Black female

Relatedly, profit was sometimes perceived as being more important to corner store owners than their customers’ needs,

*“It’s just way more profitable to sell soda and chips than healthy food.”*

—33 year old White male

The culture within stores dictated how well participants felt an app could work in this particular retail setting, given its dependency on store owner willingness to engage and interact with consumers, and potentially make changes to their store stocking, quality and pricing.

Theme 4: The App Could Help Consumers Make Informed Food Source Choices

Several key factors influenced participants’ decision-making as to where to shop for certain items. Not surprisingly, participants frequently cited cost, time and convenience as being important, and they desired an app that could enhance these factors. For example, one participant noted,

*“[The app could] definitely shorten some time down from searching a store that I don’t know what they have, or just making an assumption and then getting to the store and them not having what I want so I settle for something I don’t want.”*

—24 year old White male

In addition, another participant shared,

*“If there was an app to tell me if those stores had things like produce, I definitely would use that…like if I could go up the street to [the store] and get carrots rather than going all the way to the market… that would be more convenient.”*

—38 year old Black female

Length of residence in one’s neighborhood also played a role in participants’ knowledge of where to source certain items, and which store owners may be willing to accommodate certain needs. Familiarity with stores allowed participants to predict the quality of food products and store cleanliness, although several mentioned that having an app with crowd-sourced reviews of product and store quality would be even more beneficial.

In terms of dietary restrictions and individual food preferences, a few participants shared that they had food allergies such as gluten and dairy sensitivity, or valued eating a low-carbohydrate diet, and had trouble sourcing foods to meet those needs in their local corner stores,

*“Our family is gluten-free so that’s like kind of the big reason why we can’t do all of our shopping in [smaller] stores. It’s just like specialty diet things aren’t as accessible there or they’re just like significantly higher cost….so I think like having the benefit to search for and request products you need [in an app]…would be helpful.”*

—29 year old Asian female

Many participants agreed that dietary alternatives and fresh produce can be costly, so introducing coupons and discounts into the app would be crucial for affordability.

Desired Function and Features of the BUDConnect Interface

Theme 5 captured participant considerations and suggestions specific to the function and features of the app, which were primarily discussed during the third part of the interview process.

Theme 5: App Form and Function Suggestions

Participants generally favored the app’s user interface operability, layout and visual appeal, additionally offering suggestions for enhanced or added features that could improve its design.

Familiarity with technology played a role in participants’ likeability and level of feedback provided on the app. While some mentioned frequent and varied daily use of their smartphones, others preferred to limit their screen time or keep downloaded apps to a minimum. This led to several discussions regarding the need to prevent oversaturation of the current app market, and avoid replicating the functionality of existing apps like Yelp.

In addition, several participants voiced concern for the potential unintended consequences of the app on the local businesses being reviewed by community members, especially given opportunities to leave negative reviews or commentary,

*“The corner stores are all doing their best, and I just feel like [the app] has the opportunity to reflect poorly on them and potentially lead to less business for them, when like maybe it wasn’t their fault.”*

—32 year old White female

Similarly, another participant felt,

*“Reviews have a tendency to be more negative than positive… if the store gets a one or two star review and then people don’t go to buy collard greens [from them] which are already getting older and older then you just get into the cycle that you can’t, you know, break.”*

—33 year old White male

To mitigate this, it was proposed that BUDConnect encourage positive reviews and ratings (e.g., a clickable five-star rating system), and make photo uploads and written comments optional.

Opinions varied regarding technology’s impact on personal connections, with some praising it for its power of communication with loved ones during the COVID-19 pandemic. Others worried it may diminish face-to-face interactions. Regardless, most saw value in instant messaging between store owners and customers for in-app updates on current promotions or deals.

Visual appeal, or lack thereof, was highlighted frequently—in fact, color was often one of the first aspects mentioned when participants were asked to share their immediate reactions to seeing the initial wireframe mockup images,

*“First of all, I think that the color is really good.”*

—27 year old Asian non-binary person

*“I kind of like the color scheme of it…I’m sensitive to colors, so I like that. It was a relaxing color.”*

—35 year old Middle Eastern female

One participant noted concern that the color green in combination with the “BUD” name could be associated with the local cannabis industry, potentially discouraging community members from downloading the app if they were unfamiliar with its premise.

All participants preferred a simple, user-friendly interface, emphasizing its importance for different user types, especially older community residents.

Recommendations for enhancements to the app included linking it to existing tools such as Google Maps and the Maryland Transit Administration’s CharmPass app [30] for real-time transit information.

Related to the desire for in-app coupons and discounts, referral and rewards systems resulting in discounted healthy purchases were strongly supported by participants for themselves and their communities. They felt that earning in-app points towards reduced food costs would encourage sustained app use and store visits, therefore also benefitting the stores. However, when asked about gamifying the rewards system, views were mixed regarding whether participants could see themselves engaging in in-app games.

Finally, features like push notifications for engaging inactive users with new store updates were suggested to boost interaction and customer loyalty. This could also increase access to the app for older users:

*“Some may have a hard time trying to find that link [to the app]. So you want to make it accessible for them as well, kind of like a push notification.”*

—43 year old American Indian female

### 3.4. Revised Interface Design for Development and Testing

**Figure 2:**
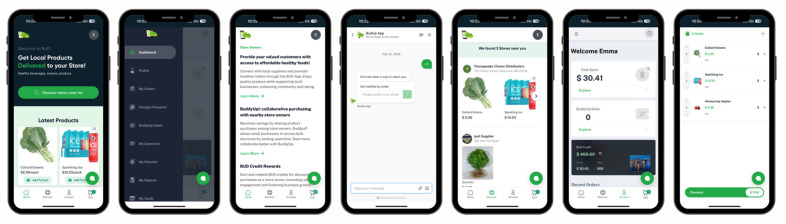
Snapshot of the current existing BUD app interface.

The research team made iterative refinements to the user interface concurrently with and subsequent to the thematic analysis. Design aspects were updated to match the current existing BUD app interface (Figure 2) for the seamless future integration of the BUDConnect module.

**Figure 3:**
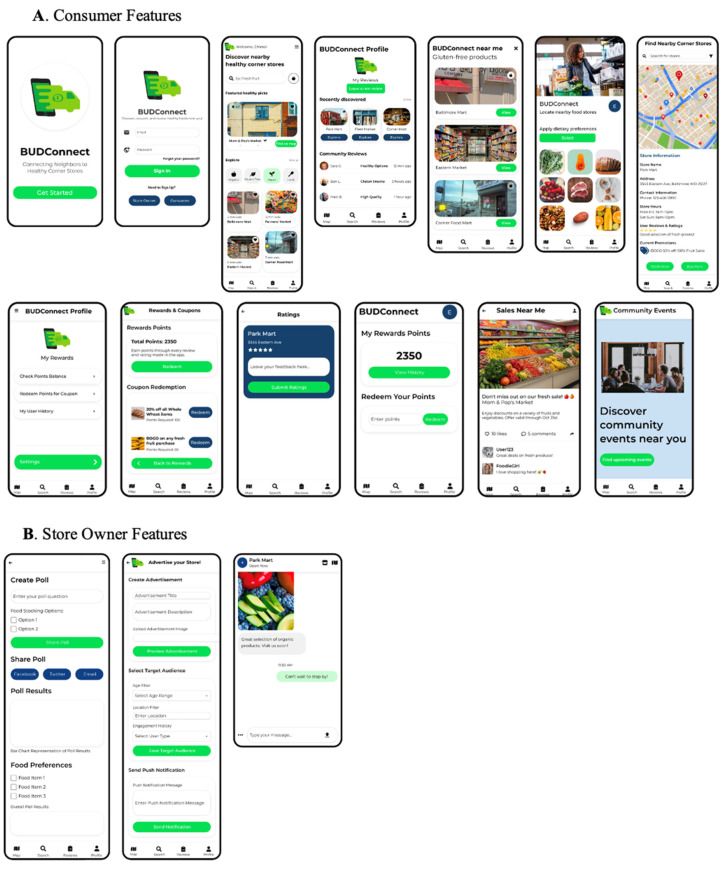
Revised BUDConnect wireframe mockups based on participant feedback.

Feature functionality was enhanced, and new features suggested by participants were built into the interface to meet the needs of both consumers (Figure 3A) and corner store owners (Figure 3B). These included a feature for consumers to filter for foods that meet specific dietary restrictions, as well as a feature for corner store owners to create advertisements and push them out to selected app users. During this process, the research team considered the feasibility of optimal back-end programming. Uizard 2024 software for UI (user interface) design [31] was used for revisions to the initial high-fidelity wireframe mockups, the result of which can be found in Figure 3.

## 4. Discussion

This is the first study to engage local community members in the interface design of a food system app that improves healthy food access in small retail settings. While the existing BUD app provides a promising solution to the supply-side challenges faced by retailers, expanding the opportunity for consumer input and communicated demand is needed. We interviewed twenty Baltimore community members who demonstrated a desire for real-time crowd-sourced information to navigate their food environments safely and effectively; interface functionality to help build community and social networks among store owners and their customers; opportunities to share positive reviews and ratings of locally owned stores and their offerings; and interoperability with other apps such as CharmPass. Rewards and referral systems resulting in the discounted purchasing of promoted healthy items were suggested to increase app use and in-store visits.

In regard to their neighborhood built environments, community members highlighted interest in enhanced in-app navigation features, such as real-time bus transportation and walking routes, which aligns with previous studies showcasing the importance of app interface design for increased user acceptability [32]. In our sample, this interest was largely related to a desire for convenience and safety when locating stores and sourcing certain products. Likewise, interviewed members of an under-resourced community in Pennsylvania highly valued convenience when sourcing food from local corner stores, especially those lacking access to a personal vehicle [33]. Providing navigation tools could decrease time spent traveling from store-to-store to acquire specific items, and in some neighborhoods, may eliminate concerns associated with crime and safety given the provision of direct routes and crowd-sourced updates depicted on an interactive map.

Our findings also shed light on the complex and nuanced task of augmenting virtual connections without compromising face-to-face interactions. This dichotomy has been researched in the context of social media and community engagement [34,35]. Apps that seek to build social relationships should do so in such a way that preserves and enhances community ties. This is especially important in retail settings where store owners may not feel like they belong to the communities their stores serve. In Baltimore and similar settings, it has been shown that store owners who lack a sense of belonging or who experience barriers to communicating with their customers tend to perceive items such as fruits and vegetables unmarketable [11,12,13,14,33]. In one San Francisco community, eleven of the seventeen interviewed small store owners reported a perceived lack of customer demand for healthy products, when, in reality, community members expressed a desire for interventions to improve the stocking of their local stores [36]. Social dynamics, such as corner store owners’ sense of community, could be strengthened by a digital BUDConnect interface that neutralizes in-person language and cultural barriers, and encourages mutual support between store owners and their customers, thereby preventing misperceptions of supply–demand.

Relatedly, community members’ emphasis on an in-app rating system that promotes positive feedback for local businesses and business owners is synchronous with recent trends in app design that emphasize community building and positive reinforcement [37]. Community members discussed considerations that align with the current literature on the need to address user inclusivity in app design, particularly for older populations [38]. Features like push notifications, large fonts and clear content mapping were suggested to enhance ease of access for those who may take longer to adopt new technology.

While notable apps targeting health behaviors and food access exist—like Goodr [39], which redirects surplus food from restaurants to food banks and shelters, and Too Good To Go [40], which connects individuals with low incomes to restaurants that sell food surplus at a reduced price—few integrate a comprehensive system that leverages community engagement and real-time functionality to accommodate the idiosyncrasies of urban food systems. In the present study, community member perspectives informed revisions to a series of initial user interface wireframe mockups. The resulting high-fidelity wireframe mockups will continue to be refined by the research team until a final user interface is reached. Front-end code will be generated in JavaScript React [41] and Tailwind CSS [42] for styling to be integrated into the existing BUD front-end framework. Once this code is embedded and flows as desired, the back-end of the app module can be programmed for full functionality and shared openly for scale-up via a GitHub repository [43].

The early engagement of community members in this design process is a noteworthy strength and could have positive implications for the app’s eventual adoption and sustained use. However, the present study was limited in several ways, including its use of snowball sampling. This method could have resulted in an unequal representation of certain perspectives, although the final sample was broadly distributed across neighborhoods and sociodemographic characteristics. While our sample size was small, the research team felt confident that data saturation was reached by the final in-depth interview. Previous studies conducting qualitative research in corner store settings have similarly reached data saturation at twenty or fewer participants [12,36]. Moreover, the findings presented here represent just one part of a complex app development process and feedback from additional key stakeholders will be considered in future stages of testing.

Finally, this work has the potential to bolster current programs and policy initiatives in Baltimore. For example, the Urban Farm Tax Credit, enacted in Maryland in 2014, gives farmers 90% off their property taxes for five years if the parcel is used for urban agriculture [44]. The BUD app and integrated BUDConnect module could help create a stable system for local farmers to sell their produce in urban settings given clear consumer demand and increased retailer revenue. Moving forward, a planned randomized controlled trial will test this digital food system intervention in multiple under-resourced urban settings, the results of which could inform future strategies targeting small store stocking requirements, local sourcing and complementary incentivization policies similar to the Urban Farm Tax Credit.

## 5. Conclusions

This study sought to engage local community members in the interface design of an app that improves healthy food access in small retail settings, especially those located far from nearby supermarkets. Opportunities for consumer input and communicated demand are crucial for digitally strengthening the urban food system supply–demand chain. Community members provided their perspectives on the overall role, function and features of the proposed design, and a series of initial high-fidelity wireframe mockups were then refined for future development and testing on a broader scale. Given its perceived acceptability and usability, the app has the potential to impact on current and future program and policy initiatives in Baltimore, including the Urban Farm Tax Credit.

## Figures and Tables

**Table 1:** Interview participant sociodemographic characteristics (*n* = 20).

Characteristic	*n*
Age (years)	
20–29	3
30–39	12
40–49	3
50–59	2
Gender	
Female	13
Male	6
Non-Binary	1
Race/Ethnicity	
Black or African American	6
White	7
Asian	2
Middle Eastern	3
American Indian	1
Hispanic or Latino	1
Food Insecure	
Yes	9
No	11
Food Assistance Program Enrollment	
SNAP	10
WIC	0
Free/Reduced-Cost School Lunch	6
Free/Reduced-Cost School Breakfast	1
Marital Status	
Married	5
Never Married	14
Divorced	1
Employment Status	
Employed	10
Unemployed	8
Disability	1
Nonprofit Sector	1
Annual Household Income	
USD 0–10k	3
USD 10–20k	5
USD 20–30k	1
USD 50–60k	1
USD 60–70k	3
USD 80k+	7

**Table 2:** Key themes (*n* = 5) and sub-themes of interview participants’ shared perspectives.

Theme	Sub-Themes
The App Could Make Food Sourcing Safer and More Successful	Urbanicity Safety and crime Available food sources
2. The App Could Help Build Community	Community cohesion Community needs
3. The App Could Support the Resurgence of “Corner Store Culture”	Store owner–customer relationships Customer expectations of store offerings Store owner support for the community
4. The App Could Help Consumers Make Informed Food Source Choices	Dietary restrictions/individual preferences Length of residence in neighborhood Cost Time and convenience Concerns over food safety and store standards
5. App Form and Function Suggestions	Familiarity with technology Cost benefit to using apps Consideration of store impact Technology hindering or facilitating personal connections Visual appeal Specific app functions

## Data Availability

The data presented in this study are available on request from the corresponding author.
